# rDNA Loci Evolution in the Genus *Glechoma* (Lamiaceae)

**DOI:** 10.1371/journal.pone.0167177

**Published:** 2016-11-21

**Authors:** Tae-Soo Jang, Jamie McCann, John S. Parker, Koji Takayama, Suk-Pyo Hong, Gerald M. Schneeweiss, Hanna Weiss-Schneeweiss

**Affiliations:** 1 Department of Botany and Biodiversity Research, University of Vienna, Rennweg 14, Vienna, Austria; 2 Cambridge University Botanic Garden, Cambridge, United Kingdom; 3 Museum of Natural and Environmental History, Shizuoka, Oya 5762, Suruga-ku, Shizuoka-shi, Sizuoka, Japan; 4 Laboratory of Plant Systematics, Department of Biology, Kyung Hee University, 1 Hoegi-Dong, Dongdaemun-Gu, Seoul, Korea; Saint Mary's University, CANADA

## Abstract

*Glechoma* L. (Lamiaceae) is distributed in eastern Asia and Europe. Understanding chromosome evolution in *Glechoma* has been strongly hampered by its small chromosomes, constant karyotype and polyploidy. Here phylogenetic patterns and chromosomal variation in *Glechoma* species are considered, using genome sizes, chromosome mapping of 5S and 35S rDNAs by fluorescence *in situ* hybridisation (FISH), and phylogenetic analyses of internal transcribed spacers (nrITS) of 35S rDNA and 5S rDNA NTS sequences. Species and populations of *Glechoma* are tetraploid (2*n* = 36) with base chromosome number of *x* = 9. Four chromosomes carry pericentric 5S rDNA sites in their short arms in all the species. Two to four of these chromosomes also carry 35S rDNA in subterminal regions of the same arms. Two to four other chromosomes have 35S rDNA sites, all located subterminally within short arms; one individual possessed additional weak pericentric 35S rDNA signals on three other chromosomes. Five types of rDNA locus distribution have been defined on the basis of 35S rDNA variation, but none is species-specific, and most species have more than one type. *Glechoma hederacea* has four types. Genome size in *Glechoma* ranges from 0.80 to 0.94 pg (1C), with low levels of intrapopulational variation in all species. Phylogenetic analyses of ITS and NTS sequences distinguish three main clades coinciding with geographical distribution: European (*G*. *hederacea*–*G*. *hirsuta*), Chinese and Korean (*G*. *longituba*), and Japanese (*G*. *grandis*). The paper presents the first comparative cytogenetic analyses of *Glechoma* species including karyotype structure, rDNA location and number, and genome size interpreted in a phylogenetic context. The observed variation suggests that the genus is still in genomic flux. Genome size, but not rDNA loci number and distribution, provides a character for species delimitation which allows better inferences of interspecific relationships to be made, in the absence of well-defined morphological differentiation.

## Introduction

Cytological plasticity plays an important role in plant diversification and speciation [1–3]. This plasticity manifests itself in changes of chromosome number (aneuploidy/dysploidy or polyploidy), chromosome structure (inversions, translocations, additions, and deletions), genome size, and more subtle changes in sequence composition [4]. Chromosome number changes have long been used to draw evolutionary and systematic inferences, but modern molecular cytogenetic techniques allow us to trace chromosomal structural changes in a phylogenetic context [5–8]. Molecular cytogenetic mapping of 5S and 35S rDNA loci using FISH (fluorescence *in situ* hybridisation) has proved useful for identifying the direction of chromosomal change in closely related species, even when chromosome numbers are conserved and chromosomes are constant in morphology [9–13].

The Eurasian genus *Glechoma* L. (Lamiaceae, Mentheae, Nepetinae) has seven species in two phylogenetic clades [14–18]. The European group [16–17] consists of widespread *G*. *hederacea* L., central European *G*. *hirsuta* Waldst. & Kit. [16] and *G*. *sardoa* (Bég.) Bég., endemic to Sardinia and Corsica [19–20]. The Asiatic group [16–18] comprises eastern Asian *G*. *longituba* (Nakai) Kuprian. and *G*. *grandis* (A.Gray) Kuprian. as well as the Chinese *G*. *biondiana* (Diels) C.Y.Wu & C.Chen, and *G*. *sinograndis* C.Y.Wu. *Glechoma sardoa*, *G*. *biondiana*, and *G*. *sinograndis* are only subtly differentiated by morphology [16] and micromorphology [15, 21–23] so only four major species—*G*. *grandis*, *G*. *hederacea*, *G*. *hirsuta*, *G*. *longituba*—are considered here.

Cytological studies of the genus *Glechoma* have been restricted to karyomorphological descriptions of classically stained mitotic metaphase chromosome complements [16, 24–25]. The chromosomes are relatively small (0.9–2.2 μm [25]) and of uniform size, and karyotypes are symmetrical. The presumptive base chromosome number is *x* = 9 although 2*n* = 36 has generally been reported, suggesting tetraploidy [25]. Support for a base number of *x* = 9 and hence tetraploidy in *Glechoma* comes from the closely related genus *Meehania*, with *M*. *montis-koyae* Ohwi and *M*. *urticifolia* (Miq.) Makino [26] both 2*n* = 18, as well as from chromosome number studies in other genera of the subtribe Nepetinae of Lamiaceae [27–31].

Chromosomal evolution in wild plant groups can be analysed in a phylogenetic context using chromosome numbers, localisation of 35S and 5S rDNA loci, and genome size variation [5, 6, 8, 32], which may allow the directionality of chromosomal evolution to be inferred [33–36]. Comparative molecular cytological analyses are lacking in *Glechoma*. Here we study patterns of genome evolution in a phylogenetic context in *Glechoma* species, as well as establishing the dynamics of rDNA and genome size evolution in these closely related, widely distributed, polyploid taxa, using FISH and flow cytometry. The specific aims of this study are (1) to assess phylogenetic relationships of multiple populations of taxa based on ITS and 5S rDNA NTS sequences, (2) to elucidate trends in evolution of 5S and 35S rDNA in the genus, and test whether these correlate with phylogenetic relationships between the polyploids, and (3) to examine patterns of genome size change in *Glechoma*.

## Materials and Methods

### Ethics Statement

The investigated taxa are neither endangered nor protected. All material was collected at public land, where no special permissions are required.

### Plant material

A total of 48 individuals, two to six individuals per population, of four species of *Glechoma* and three individuals of one population of *Meehania urticifolia* were collected from natural populations and transplanted to the Botanical Garden of the University of Vienna (Table 1).

**Table 1:** Plant materials, accession numbers (acc.), localities, detailed voucher information, genome size (1C and 2C values in picograms) and number of ribosomal DNA signals in species of the genus *Glechoma* and *Meehania urticifolia*.

Taxa	acc.	Locality; Voucher	2C ± S.D.	1C	rDNA signals		Type	GenBank accession numbers	
					35S	5S		ITS	5S-NTS
*Glechoma grandis* (A.Gray) Kuprian.	1	Japan, Tottori; Ikeda *et al*., 2–1	1.850 ± 0.006	0.93	8 (4)	4	(i)	–	–
	1	Japan, Tottori; Ikeda *et al*., 2–2	1.774 ± 0.005	0.89	8 (4)	4	(i)	–	–
	1	Japan, Tottori; Ikeda *et al*., 2–4	1.820 ± 0.002	0.91	–	–		–	–
	2	Japan, Tokyo; Takayama, 3–6	1.875 ± 0.004	0.94	6 (2)	4	(iii)	KX351269	
	2	Japan, Tokyo; Takayama, 3–7	1.869 ± 0.009	0.93	6 (2)	4	(iii)	KX351268	KX351248– KX351250
	2	Japan, Tokyo; Takayama, 3–8	1.843 ± 0.001	0.92	–	–		–	–
*G*. *hederacea* L.	1	Latvia, Lilaste; Jang, 818	1.647 ± 0.001	0.82	8 (4)	4	(i)	KX351258	KX351239– KX351241
	1	Latvia, Lilaste; Jang, 819	1.643 ± 0.001	0.82	8 (4)	4	(i)	–	–
	1	Latvia, Lilaste; Jang, 820	1.654 ± 0.002	0.83	–	–		KX351259	–
	1	Latvia, Lilaste; Jang, 821	1.655 ± 0.002	0.83	–	–		–	–
	1	Latvia, Lilaste; Jang, 822	1.662 ± 0.008	0.83	–	–		–	–
	1	Latvia, Lilaste; Jang, 823	1.655 ± 0.001	0.83	–	–		–	–
	2	Austria, Vienna; Jang, 321	1.653 ± 0.007	0.83	–	–		KX351255	–
	2	Austria, Vienna; Jang, 322	1.642 ± 0.004	0.82	6 (4)	4	(v)		–
	2	Austria, Vienna; Jang, 323	1.645 ± 0.007	0.82	6 (4)	4	(v)	KX351256	KX351233– KX351235
	2	Austria, Vienna; Jang, 325	1.651 ± 0.001	0.83	–	–		KX351257	–
	3	Austria, Perchtoldsdorf; Jang, 13	1.629 ± 0.004	0.81	–	–		–	–
	3	Austria, Perchtoldsdorf; Jang, 14	1.625 ± 0.005	0.81	7 (4)	4	(iv)	KX351261	KX351236– KX351238
	3	Austria, Perchtoldsdorf; Jang, 15	1.647 ± 0.002	0.82	–	–		–	–
	3	Austria, Perchtoldsdorf; Jang, 16	1.596 ± 0.002	0.80	–	–		KX351262	–
	4	Poland, Katowice; Jang, 925	1.626 ± 0.002	0.81	7 (3)	4	(ii)	KX351260	–
	4	Poland, Katowice; Jang, 926	1.621 ± 0.006	0.81	–	–		–	–
	4	Poland, Katowice; Jang, 927	1.621 ± 0.001	0.81	–	–		–	–
*G*. *hirsuta* Waldst. & Kit.	1	Austria, Leopoldsberg; Jang, 511	1.638 ± 0.001	0.82	–	–		KX351263	–
	1	Austria, Leopoldsberg; Jang, 512	1.639 ± 0.009	0.82	7 (3)	4	(ii)	KX351264	–
	1	Austria, Leopoldsberg; Jang, 513	1.631 ± 0.001	0.82	7 (3)	4	(ii)	KX351265	KX351227– KX351229
	1	Austria, Leopoldsberg; Jang, 514	1.643 ± 0.003	0.82	–	–		–	–
	1	Austria, Leopoldsberg; Jang, 515	1.639 ± 0.002	0.82	–	–		–	–
	2	Austria, Perchtoldsdorf; Jang, 7	1.659 ± 0.008	0.83	–	–		–	–
	2	Austria, Perchtoldsdorf; Jang, 8	1.668 ± 0.008	0.83	–	–		KX351266	–
	2	Austria, Perchtoldsdorf; Jang, 9	1.655 ± 0.010	0.83	7 (4)	4	(iv)	–	–
	2	Austria, Perchtoldsdorf; Jang, 10	1.645 ± 0.001	0.82	10 (4)	4	(iv)	KX351267	KX351230– KX351232
	2	Austria, Perchtoldsdorf; Jang, 11	1.678 ± 0.012	0.84	7 (4)	4	(iv)	–	–
	2	Austria, Perchtoldsdorf; Schneeweiss, 52	1.688 ± 0.003	0.84	–	–		–	–
	2	Austria, Perchtoldsdorf; Schneeweiss, 53	1.698 ± 0.018	0.85	–	–		–	–
	2	Austria, Perchtoldsdorf; Schneeweiss, 54	1.675 ± 0.006	0.83	–	–		–	–
*G*. *longituba* (Nakai) Kuprian.	1	Korea, Gwacheon; Hong, 310	1.793 ± 0.003	0.90	8 (4)	4	(i)	–	–
	1	Korea, Gwacheon; Hong, 311	1.810 ± 0.006	0.91	–	–		–	–
	2	Korea, Munsan; Jang, 8415	1.805 ± 0.004	0.90	8 (4)	4	(i)	KX351251	–
	2	Korea, Munsan; Jang, 8416	1.803 ± 0.003	0.90	–	–		–	–
	2	Korea, Munsan; Jang, 8417	1.804 ± 0.001	0.90	8 (4)	4	(i)	KX351252	KX351242– KX351244
	2	Korea, Munsan; Jang, 8418	1.846 ± 0.001	0.92	8 (4)	4	(i)	–	–
	3	China, Hubei; Xi, 01	1.841 ± 0.003	0.92	8 (4)	4	(i)	KX351253	KX351245– KX351247
	3	China, Hubei; Xi, 02	1.881 ± 0.016	0.94	–	–		–	–
	3	China, Hubei; Xi, 03	1.873 ± 0.010	0.94	8 (4)	4	(i)	–	–
	3	China, Hubei; Xi, 04	1.844 ± 0.004	0.92	–	–		–	–
	3	China, Hubei; Xi, 05	1.849 ± 0.002	0.92	–	–		–	–
	3	China, Hubei; Xi, 06	1.830 ± 0.005	0.92	8 (4)	4	(i)	KX351254	–
*Meehania urticifolia* (Miq.) Makino	1	Korea, Gapyeong; Jang, 04241	1.162 ± 0.002	0.58	4 (2)	2		KX351270	–
	1	Korea, Gapyeong; Jang, 04242	1.164 ± 0.002	0.58	4 (2)	2			–
	1	Korea, Gapyeong; Jang, 04243	1.163 ± 0.002	0.58	–	–			–

Note: S.D.: standard deviation, (): co-localized with 5S rDNA sites,–: not studied; type (i) to (v) was distinguished by the number and location of rDNA sites (see text).

### Chromosome numbers and fluorescence *in situ* hybridisation (FISH)

Actively growing root-tips were pretreated with 0.002 M solution of 8-hydroxyquinoline for 2.5 h at room temperature and 2.5 h at 4°C, then fixed in ethanol: acetic acid (3: 1) and stored at –20°C until use. *Glechoma hederacea* and *G*. *longituba* have a gynodioecious sexual system comprising females and hermaphrodites [37–38]. Only hermaphrodites were examined. Chromosome numbers were initially determined by standard Feulgen staining of root tip cells [6]. To improve resolution of karyotype structure, preparations were also made using enzymatic digestion (described below), stained with 2ng/μl DAPI (4’,6-diamidino-2-2phenylindole), mounted in antifade medium Vectashield (Vector Laboratories, Burlingame, CA, USA) and examined under a fluorescence microscope.

Chromosomes for FISH were prepared by enzymatic digestion and squashing as described in Jang and Weiss-Schneeweiss [39]. Briefly, meristematic root cells were digested with 1% cellulose Onozuka (Serva, Heidelberg, Germany), 1% cytohelicase (Sigma-Aldrich, Vienna, Austria), and 1% pectolyase (Sigma-Aldrich, Vienna, Austria), and squashed in 60% acetic acid. Cover slips were removed at –80°C and preparations air-dried.

Probes used for FISH were complete coding regions of 25S rDNA and 18S rDNA from *Arabidopsis thaliana* in plasmids pSK^+^ (to detect 35S rDNA loci; sequences including those regions originally under GenBank accession numbers X16077 and X52320) and the genic region of 5S rDNA from *Prospero autumnale* in plasmid pGEM-T easy [6]. Probes were labeled with biotin-16-dUTP or digoxygenin-11-dUTP (Roche, Vienna, Austria) by PCR (5S rDNA) or using a nick translation kit (35S rDNA; Roche, Vienna, Austria). Formamide-free *in situ* hybridisation was performed as described earlier [39]. Digoxygenin- and biotin-labeled probes were detected using antidigoxygenin-FITC (5 μg/mL: Roche, Vienna, Austria) and ExtrAvidin-Cy3, respectively (2 μg/mL: Sigma-Aldrich, Vienna, Austria).

Chromosomes were examined with an AxioImager M2 epifluorescent microscope, photographed with a high resolution black-white microscopy camera (Carl Zeiss, Vienna, Austria), and files processed using AxioVision 4.8 (Carl Zeiss, Vienna, Austria) with only those functions that apply to whole images equally. At least 15–20 well-spread metaphases and prometaphases were analysed for each individual. One to three individuals per population, in total 22 individuals of four species of *Glechoma* and two individuals of *Meehania urticifolia*, were analysed using FISH (Table 1).

### Genome size estimation

Genome sizes of 48 plants of *Glechoma* and three plants of *Meehania urticifolia* were determined by flow cytometry with *Solanum pseudocapsicum* (1C = 1.29 pg/1C [6, 40]) as internal standard. Freshly collected leaves from individual plants and the standard were chopped together in Otto’s buffer I [41], filtered through a nylon mesh, and incubated with RNase at 37°C in a water-bath for 30 min. Propidium iodide (PI) staining was performed in Otto’s buffer II at 4°C. Each individual was measured three times. Measurements were performed with a CyFlow flow cytometer (Partec, Germany) equipped with a green laser (532 nm, Cobolt, Sweden). The 1C values were calculated according to the assumed linear fluorescence intensity relationship of both object and standard nuclei [6, 40].

### DNA sequencing and phylogenetic analyses

Total genomic DNA was extracted from silica gel-dried leaf material of selected analysed individuals (Table 1), as described in Jang et al. [6]. The internal transcribed spacer (ITS) region of nuclear ribosomal DNA was amplified and sequenced using universal ITS primers [6; ITS_18s_F: 5′-ACCGATTGAATGGTCCGGTGAAGTGTTCG-3′ and ITS_26s_R: 5′-CTGAGGACGCTTCTCCAGACTACAATTCG-3′; Sigma-Aldrich, Austria]. The non-transcribed spacer region of the 5S rDNA repeat was amplified using the primers reported in Weiss-Schneeweiss et al. [5; 5S_fwd: 5’-AGTTAAGCTTTGGGCGAGAGTA and 5S_rev: 5’-AGTTCTGATGGAATTCGGTGYTKT; Sigma Aldrich, Austria].

Briefly, polymerase chain reactions consisted of 1 × buffer (MBI Fermentas, St Leon-Rot, Germany), 2.5 mM MgCl_2_ (MBI Fermentas), 2 μM of each of the dNTPs (MBI Fermentas), 0.1 μM of each primer (Sigma Aldrich, Vienna, Austria), and 2.5 U of RedTaq polymerase (Sigma Aldrich). The cycling conditions included initial denaturation at 96°C for 1 min, followed by 35 cycles each of 1 min at 96°C, 30 sec at 55°C, 45 sec at 72°C, and final elongation at 72°C for 10 min. Amplified fragments were separated on a 1.5% agarose gel, and PCR products were purified from the gel using Invisorb^®^ Spin DNA Extraction Kit (Invitek GmbH, Berlin, Germany). PCR amplification products of the 5S rDNA non-transcribed spacer DNA were cloned as described in Emadzade et al. [42] with three clones sequenced per individual and one individual per species (Table 1).

Sequences were assembled in SeqManII 5.05 (Lasergene, Madison, WI) and manually aligned using BioEdit 7.0.5.3 [43]. Model testing and inference of phylogenetic relationships were performed using maximum likelihood (ML) analysis as implemented in IQ-TREE 0.9.5 [44]. Gaps were treated as unknown characters. The best model was chosen according to the Bayesian Information Criterion and used for the final ML analysis. Nodal support was determined using non-parametric bootstrapping [45] with 1,000 replicates. All sequences were deposited in GenBank (Table 1). Trees obtained from ITS sequences were rooted using *Meehania urticifolia* as outgroup, those obtained from NTS sequences were rooted using paralogy rooting, i.e., sequences from one monomer type (see Result) are used as outgroup for those from the other monomer type.

## Results

### Chromosome numbers

**Fig 1:**
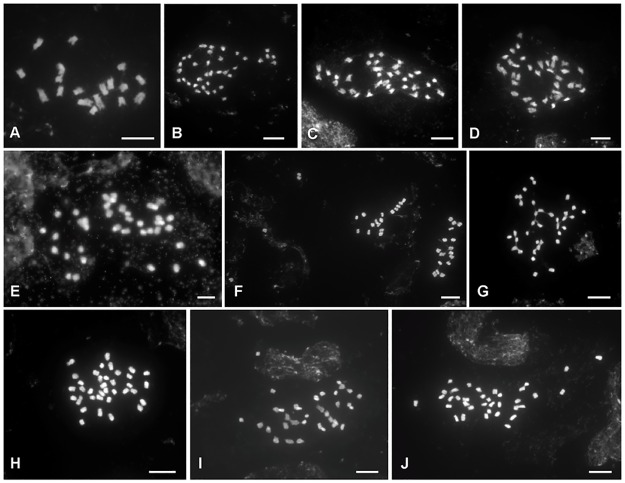
Mitotic metaphase chromosome plates of *Meehania urticifolia* and *Glechoma* species stained with DAPI. **(A)** *Meehania urticifolia* (Gapyeong, Korea). **(B)** *Glechoma grandis* (Tottori, Japan). **(C)** *G*. *hederacea* (Perchtoldsdorf, Austria). **(D)** *G*. *hederacea* (Katowice, Poland). **(E)** *G*. *hederacea* (Vienna, Austria). **(F)** *G*. *hederacea* (Lilaste, Latvia). **(G)** *G*. *hirsuta* (Leopoldsberg, Austria). **(H)** *G*. *hirsuta* (Perchtoldsdorf, Austria). **(I)** *G*. *longituba* (Hubei, China). **(J)** *G*. *longituba* (Munsan, Korea). Scale bars = 5 um.

All 48 plants of four *Glechoma* species from nine populations, at least two individuals per species, were tetraploids with 2*n* = 36. Three plants of *Meehania urticifolia* were diploid with 2*n* = 18 (Table 1; Fig 1).

### Numbers and distribution patterns of ribosomal DNA loci

Twenty four plants, representing one to three individuals per population of each species of *Glechoma* and *Meehania*, were examined by FISH.

**Fig 2:**
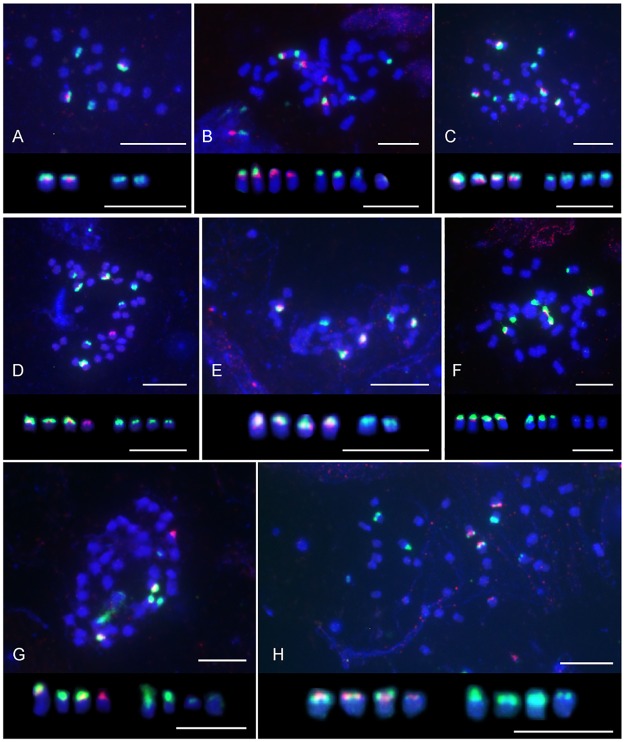
Karyotypes and localization of 35S (green) and 5S (red) rDNA loci in *Meehania urticifolia* and *Glechoma* species. **(A)** *Meehania urticifolia* (Gapyeong, Korea). **(B)** *Glechoma grandis* (Tokyo, Japan). **(C)** *G*. *hederacea* (Lilaste, Latvia). **(D)** *G*. *hederacea* (Katowice, Poland). **(E)** *G*. *hederacea* (Vienna, Austria). **(F)** *G*. *hederacea* (Perchtoldsdorf, Austria). **(G)** *G*. *hirsuta* (Leopoldsberg, Austria). **(H)** *G*. *longituba* (Gwacheon, Korea). Scale bar = 5 μm.

In *M*. *urticifolia*, one 5S rDNA locus and two 35S rDNA loci were found. The 5S rDNA locus was in the same chromosomal arm as, and proximal to, one of the 35S rDNA loci. The second 35S rDNA locus was in the subterminal region of the short arm of a different chromosome (Table 1; Fig 2A).

**Fig 3:**
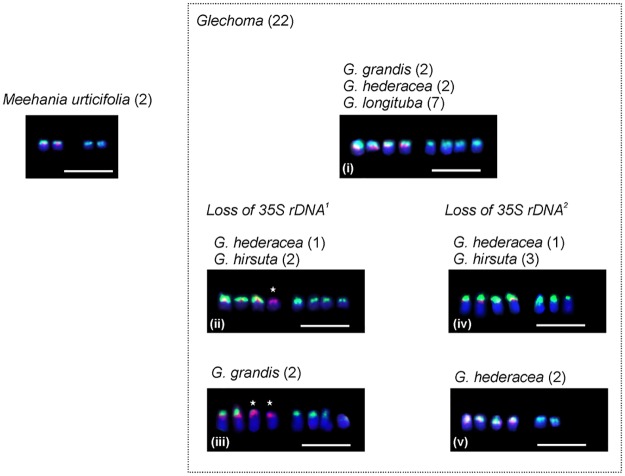
Summary of rDNA site number variation in *Meehania urticifolia* and *Glechoma*. Loss of 35S rDNA sites inferred from a doubling of the *Meehania* pattern and seen in *G*. *grandis*, *G*. *hederacea*, *G*. *longituba* (i) is indicated by stars. ^1^: Loss of 35S rDNA from chromosomes carrying 5S rDNA. ^2^: Loss of 35S rDNA from chromosomes without 5S rDNA. Roman numerals (i–v) indicate the five distribution types of rDNA (see Results). Number of individuals studied in brackets. Scale bar = 5 μm.

*Glechoma* consistently possessed four 5S rDNA signals. The number of 35S rDNA sites varied from six to eight, but in one individual of *G*. *hirsuta* three further very weak signals were seen on three different chromosomes (Table 1; Fig 2F). 5S rDNA sites were always located on short arms and in more proximal positions than the 35S rDNA loci (Fig 3). 35S rDNA occupied subterminal regions of the short arms (Fig 2) and occasionally could be seen as distinct secondary constrictions (Fig 2F and 2G).

Five rDNA distribution types were observed in the 22 *Glechoma* plants (Table 1; Figs 2B–2H and 3):

1. four chromosomes with both 35S and 5S rDNA signals on short arms, with four other chromosomes carrying subterminal short-arm 35S rDNA (Figs 2C, 2H and 3). This pattern was seen in two *G*. *grandis* plants from Tottori, Japan, two *G*. *hederacea* plants from Latvia, and seven individuals in three populations of *G*. *longituba* from China and Korea.
2. three chromosomes with 35S and 5S rDNA subterminal on short arms, one chromosome with a short-arm 5S rDNA locus, and four chromosomes with subterminal short-arm 35S rDNA sites (Figs 2D, 2G and 3). This pattern was seen in a *G*. *hederacea* plant from Poland and two *G*. *hirsuta* plants from an Austrian population.
3. two chromosomes with terminal short-arm 35S and 5S rDNA, two chromosomes with a short-arm 5S rDNA site, and four chromosomes with subterminal short-arm 35S rDNA loci (Figs 2B and 3). Two plants of *G*. *grandis* from Tokyo showed this pattern.
4. four chromosomes with short-arm subterminal 35S and 5S rDNA signals, with three other chromosomes carrying short-arm 35S rDNA sites (Figs 2F and 3). One plant of *G*. *hederacea* and two of *G*. *hirsuta*, all from Perchtoldsdorf in Austria (Table 1), had this pattern, while a single *G*. *hirsuta* plant showing this pattern had additional, weak, short-arm pericentric 35S rDNA signals on three different chromosomes (Fig 2F).
5. four chromosomes with short-arm 35S and 5S rDNA signals, with two other chromosomes carrying short-arm subterminal 35S rDNA signals (Figs 2E and 3). This was observed in two *G*. *hederacea* plants from Vienna.

### Genome sizes

The 1C DNA content of *Glechoma* differs between European *G*. *hederacea* and *G*. *hirsuta* (0.82 pg and 0.83 pg) and the Asian species *G*. *grandis* and *G*. *longituba* (0.92 pg and 0.91 pg). The genome size of *Meehania urticifolia* was 0.58 pg (Table 1).

### Phylogenetic relationships in *Glechoma*

ITS sequences of 19 plants of one to three individuals per population of each *Glechoma* species were analysed. The length of the ITS region ranged from 597 to 607 bp. The total alignment was 608 bp long, with 357 variable characters, of which 153 were parsimony-informative.

**Fig 4:**
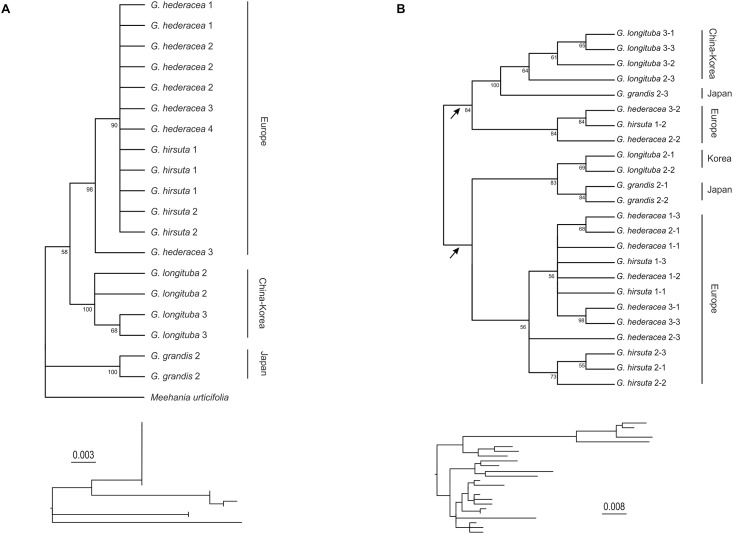
Phylogenetic trees derived from maximum likelihood analysis of ITS (A) and 5S rDNA NTS (B) sequences. Shown are cladograms (above) with bootstrap support values > 50% and, below, phylograms (topology only) with scale bars (substitutions per site). Numbers after species names refer to different accessions (Table 1) and to clone numbers (after dash). Arrows in **(B)** indicate two monomer types.

*Glechoma grandis* and *G*. *longituba* formed monophyletic well-supported clades (bootstrap support, BS 100%; Fig 4A). Within *G*. *longituba*, two subclades were identified—Chinese (BS 68%), and Korean (BS 59%). *Glechoma longituba* was recovered as sister group (BS 58%) to the clade comprising *G*. *hederacea* and *G*. *hirsuta* (BS 98%). Relationships between the latter two species, however, remain unresolved (Fig 4A).

In addition, the non-transcribed spacer region (NTS) of 5S rDNA was cloned in a subset of individuals (one to three per species) for which ITS sequences were available. For each individual three clones were sequenced (Table 1). In most individuals two distinct NTS sequence types were identified, as expected after a duplication of 5S rDNA loci following polyploidization. Fragment length ranged from 453 to 513 bp with NTS alignment spanning 517 bp. 118 characters were variable, of which 52 were parsimony-informative. The maximum likelihood tree includes a split into two groups (BS 84%) corresponding to the two monomer types, each containing clones of all species (Fig 4B). Although the number of sequences per species differed between the two groups, inferred relationships among species within each group were congruent. Thus, *G*. *grandis* and *G*. *longituba* were sister taxa within a clade (BS 83–100%) that was itself a sister group to a clade comprising *G*. *hederacea* and *G*. *hirsuta* (BS 56–84%; Fig 4B).

## Discussion

Interpreting variation in rDNA loci number and localisation in closely related species within a phylogenetic framework and in an evolutionary context has provided valuable information about relationships between diploids and polyploids when genetic knowledge is limited (e.g., *Prospero* [6]; *Aeschynomene* [7]; *Hepatica* [9]; *Heliophila* [34]; *Nicotiana* [46]). The *Glechoma* species investigated here show micro- and macromorphological similarity, a uniform basic chromosome number and the same level of polyploidy [15–16, 21, 23]. However, the species exhibit variation in the number and distribution of rDNA loci and in their genome sizes.

### Chromosome numbers and genome size variation

In *Meehania* the base number is *x* = 9 [24–26, 47]. *Meehania* species are either exclusively diploid (*M*. *montis-koyae*: 2*n* = 18 [26]) or contain both diploids and triploids (*M*. *urticifolia*: 2*n* = 18 and 27 [47]). Nearly all chromosome number reports for *Glechoma*, across its Eurasian range indicate tetraploidy (2*n* = 4*x* = 36) with a few pentaploids and hexaploids in *G*. *hederacea* and *G*. *grandis* [25, 48]. Diploids with 2*n* = 18 have been reported sporadically in Norway, Finland, and Russia, although usually without localities [49–51]. Polyploidisation then seems to have been early in the evolution of the genus, perhaps from a *Meehania*-like diploid ancestor. All *Glechoma* species studied here possess a uniform karyotype of metacentrics and submetacentrics.

Genome size values, the first estimates for the genus *Glechoma* and *Meehania urticifolia*, range from 0.58 to 0.94 pg per 1C (Table 1). *Glechoma* C-values are larger than those in other genera of the subtribe Nepetinae of Lamiaceae—*Nepeta teydea* 2*n* = 2*x* = 16, 0.28 pg per 1C [52], *Hyssopus officinalis x* = 6 (but of unknown ploidy level), 0.6 pg [53–54], and *Meehania urticifolia* 2*n* = 2*x* = 18, 0.58 pg (this study). In addition, the eastern Asian taxa of *Glechoma* have slightly higher C-values than European taxa (Table 1), coinciding with their phylogenetic distinctness (Fig 4).

### rDNA evolution and phylogenetic relationships in *Glechoma*

Most frequently in *Glechoma* there are eight 35S rDNA sites and four 5S rDNA sites, all in subterminal positions on short arms, as observed in *G*. *grandis*, *G*. *hederacea* and *G*. *longituba* (Fig 3). This is additive with respect to diploid *Meehania urticifolia* (Fig 3) which may represent the ancestral condition of *Glechoma*. A further four rDNA distribution patterns in *Glechoma* can be derived by loss of one of the 35S rDNA loci in *Meehania*, although never of both (Fig 3). A similar phenomenon has been reported in *Paphiopedilum* section *Parvisepalum* in Orchidaceae [12]. One individual of *G*. *hirsuta* had three additional, albeit very weak, signals of 35S rDNA in pericentric positions on chromosomes without other rDNA signals. Interestingly, all *Glechoma* species, except *G*. *longituba* (seven individuals) exhibit between-individual variation in their 35S rDNA patterns. By contrast, the number and the distribution of 5S rDNA signals is constant. Greater polymorphism of 35S rDNA in comparison to 5S rDNA is common in plants [9, 55–59]. The close proximity of these two genes within the same chromosome, or even chromosome arm, also characterises many other plant groups [60].

Phylogenetic relationships inferred from ITS and 5S rDNA NTS sequences (Fig 4) are congruent with previous molecular phylogenetic results reported for the genus [21, 61] in that the distinctness of Asian *G*. *grandis* and *G*. *longituba* is well supported, while the European *G*. *hirsuta* and *G*. *hederacea* are intermixed (Fig 4) reflecting the high degree of morphological similarity and taxonomic uncertainty in this species pair. *Glechoma* is a relatively young genus (3–6 mya [61–62]) and contains possible hybrids and several ploidy levels [48]. Coincident low levels of rDNA locus variation, particularly of 35S rDNA, coupled with low amounts of morphological differentiation have been reported in other polyploid complexes [7, 55].

Phylogenetic analyses of the 5S rDNA NTS regions suggest the presence of two types of monomers in every *Glechoma* species (Fig 4B), a pattern congruent with an allopolyploid origin. The alternative scenario of an autopolyploid origin is less likely, as it would require divergent evolution of homologous 5S rDNA loci, which has not been reported for plants so far. However, to establish what role hybridisation has played in the origin and evolution of the genus *Glechoma* needs other, more variable, sequences to be analysed, coupled with more extensive sampling. Polyploidisation has frequently been implicated in diversification and the colonisation of new habitats and areas [63–64]. Thus, colonisation and spread across Eurasia by the genus *Glechoma* might have been facilitated polyploidy [48].

## Conclusions

This is the first molecular cytogenetic analysis of karyotype structure and genome size variation in the tetraploid genus *Glechoma*. An evolutionary interpretation in a phylogenetic context has been possible. 35S rDNA locus number is variable within and between *Glechoma* species, with several patterns shared by different species and with nearly every species exhibiting more than one pattern. The ancestral karyotype of *Glechoma* remains unidentified. However, plants carrying four 5S rDNA and eight 35S rDNA sites represent a doubling of the pattern seen in the closely related diploid *Meehania urticifolia* which may be a candidate for the structure of the ancestral karyotype. The variability of 35S rDNA sites suggests that the polyploid genomes in *Glechoma* are still in genomic flux [65]. Genome sizes and rDNA locus variation might provide characters to support species delimitation within *Glechoma*, since morphological diagnostic features are particularly weak [15–16, 21–23].
